# Bioacoustic classification of avian calls from raw sound waveforms with an open-source deep learning architecture

**DOI:** 10.1038/s41598-021-95076-6

**Published:** 2021-08-03

**Authors:** Francisco J. Bravo Sanchez, Md Rahat Hossain, Nathan B. English, Steven T. Moore

**Affiliations:** 1 School of Engineering and Technology, Central Queensland University, North Rockhampton, QLD Australia; 2 School of Health, Medical and Applied Sciences, Flora, Fauna and Freshwater Research, Central Queensland University, Townsville, QLD Australia

**Keywords:** Machine learning, Conservation biology

## Abstract

The use of autonomous recordings of animal sounds to detect species is a popular conservation tool, constantly improving in fidelity as audio hardware and software evolves. Current classification algorithms utilise sound features extracted from the recording rather than the sound itself, with varying degrees of success. Neural networks that learn directly from the raw sound waveforms have been implemented in human speech recognition but the requirements of detailed labelled data have limited their use in bioacoustics. Here we test SincNet, an efficient neural network architecture that learns from the raw waveform using sinc-based filters. Results using an off-the-shelf implementation of SincNet on a publicly available bird sound dataset (NIPS4Bplus) show that the neural network rapidly converged reaching accuracies of over 65% with limited data. Their performance is comparable with traditional methods after hyperparameter tuning but they are more efficient. Learning directly from the raw waveform allows the algorithm to select automatically those elements of the sound that are best suited for the task, bypassing the onerous task of selecting feature extraction techniques and reducing possible biases. We use publicly released code and datasets to encourage others to replicate our results and to apply SincNet to their own datasets; and we review possible enhancements in the hope that algorithms that learn from the raw waveform will become useful bioacoustic tools.

## Introduction

The study of animal vocalisations and sounds, bioacoustics, is a field of active research that supports wildlife monitoring, management and conservation^1^ through the identification of target species from field recordings. Advances in digital sound recording hardware and storage have led to the widespread use of autonomous recording units. These are digital sound recorders that, with small servicing requirements, are deployed in the field for weeks to months (or indefinitely) and acquire large amounts of acoustic data. This passive acoustic monitoring (PAM) is a valuable tool for wildlife conservationists and managers because it can target large spatial areas and long time scales at a fraction of the cost of traditional survey methods^2^. PAM targets broad animal groups such as insects, frogs, birds, microbats and marine mammals^3^. Additional benefits of the technique include: minimal disturbance from observers which aids in the detection of shy species^4^; being deployable in ecosystems that may be logistically difficult to survey^5^; the ability to collect data during unfavourable conditions or times of the day or year^6,7^; reductions in observer bias^6^; and the generation of permanent, objective records^6,8^ that may be further analysed by alternative or newer techniques. PAM data can be used to detect and monitor rare or inconspicuous species^6^, study animal behaviour^9^, assess wildlife populations^5,10^, or track spatial and temporal population changes^4,9,11^.

The drawback to PAM is that manual processing of audio recordings by experts requires substantial effort^4^. It cannot realistically be done when replication across geographic and temporal scales results in thousands of hours of sound recordings^3^, or when scaling up to permanent recording stations^12^. The key challenge in the field is the ability to convert the large volume of acoustic records into usable data. Ideally, software trained to recognise the calls of one or multiple target species would process the acoustic data and provide timestamps of target calls in the recording sequence^6^. There have been significant advances in the development of software to process acoustic data and identify species^1^, and this software generally matches specific sound signatures or trains machine learning algorithms to recognise one or multiple species. However, software is still not at a level where general-purpose tools are able to identify species from real-life field recordings without significant user input^1,13,14^.

Processing bioacoustic data comes with inherent challenges^1,8^; overlapping target sounds, environmental and background noises, power variance of sound due to varying distances between source and recorder, and the variability of the sound even within the same species. Another challenge is the lack of adequately labelled datasets to train software. There are large and growing repositories of bioacoustic sounds, particularly birdsong (Macaulay Library, Tierstimmenarchiv, Xeno-canto)^13^. However, training best-performing machine learning algorithms requires detailed labelling, which is generally lacking in these large datasets^13,15,16^.

There are two key elements in the recognition of a bioacoustic sound: the detection of the acoustic event; and its classification into, for example, the vocalisation of a known species. These two elements may be attempted at the same time or as distinct tasks^16^. Our research focuses on the second element, classification. Matching a sound against a set of reference sounds is a typical supervised machine learning task. Labelled sounds in a training dataset allow the algorithm to learn and make predictions for new sounds^17^. Researchers have used a variety of machine learning algorithms such as Hidden Markov Models, Gaussian Mixture Models and Support Vector Machines^18^. More recently the focus has shifted toward the use of deep learning methods such as Convolutional Neural Networks (CNN)^7^.

Machine learning algorithms in bioacoustics are generally trained on features extracted from the sound, as training directly on the sound has not been practical^19^. Digital sound recorders convert a pressure sound wave into a digital representation. In broad terms a digital recording (*i.e.* raw waveform), is a series of bits corresponding to the magnitude of the pressure wave for a given sample rate (number of inputs per time) and bit depth (number of bits per data point). In practical terms the raw waveform is useful for storage, playback and further processing. But the high dimensionality of the raw waveform has limited its direct usage in machine learning, the so-called: “curse of dimensionality”^1^. Traditionally, machine learning algorithms are trained on extracted features, typically spectrogram-like representations of the sound^19^. Conversions, including fast Fourier transform, short-time Fourier transform, linear prediction coefficients, wavelets and chirplets^19^, reduce the dimensionality of the sound to facilitate the machine learning process. These may be further processed into handcrafted features, such as Mel frequency cepstral coefficients, that transform the linear frequency according to a perceptual scale (Mel scale) reflecting the non-linear human perception of sound^20^. The popular use and relative success of the Mel scale in bioacoustics is being progressively challenged by researchers, as the use of the acoustic spectrum by other animals is likely to differ from that derived from human perception^18,19^.

An alternative that may be particularly useful in bioacoustics is training directly from the raw waveform, where algorithms autonomously select the relevant elements of the sound. Human speech researchers are developing deep-learning algorithms trained on the raw waveform. Due to the problem of high dimensionality, early examples involved significant amounts of labelled data (over 200 h of speech)^21^. More recently researchers have successfully trained speech-recognition algorithms using datasets of moderate size (a few hours), matching the performance of other state-of-the-art approaches^22^. Training an algorithm directly on the raw waveform bypasses the feature extraction step altogether and allows the algorithm to select those elements of the sound that best match the required task. This has significant implications for processing human speech, leading researchers to claim that ‘*in the same way deep architectures changed the landscape of computer vision by directly learning from raw pixels, we believe that future end-to-end speech recognition systems will learn directly from the waveform*’^22^. In the field of bioacoustics, the selection of extracted features is a complex step as there are numerous possibilities but none established as ideal, are often borrowed from other fields (e.g. human speech processing) and their performance may vary between tasks^1^. Extracted feature selection is critical for performance and may involve trial and error, some level of automation^18^, or may just follow previous successes possibly impacting performance when applied to new tasks or datasets. Training directly from the raw waveform bypasses this step, allowing the algorithm to select automatically the elements of the sound best suited for the task that could otherwise be lost at the feature selection or extraction step^23^. Despite its potential, and probably due to their perceived lack of efficiency^24^, we could only find two examples^25,26^ of bioacoustic researchers using the raw waveform to train a machine learning algorithm.

This study utilised a published open-source CNN architecture^27^ (SincNet), designed to process raw human-speech samples, to identify species from raw digital waveforms sourced from a publicly-released and richly annotated birdsong dataset^15^ (NIPS4Bplus). We compare the performance of enhanced SincNet models that rely on sinc-based filters, against a similar architecture that learns directly from the raw waveform without these filters, and against standard techniques using pre-trained models on transformed sounds and extracted features.

The first convolutional layer of a standard CNN processing the raw waveform deals with a high dimensionality input and is very susceptible to a known issue; the vanishing gradient problem^27^. SincNet constrains the shape of the first convolutional layer with a series of band-pass filters based on the cardinal-sine or sinc function. For each filter the CNN learns from the training data only the low and high cut-off frequencies, thus requiring fewer parameters than standard CNNs and helping SincNet to be more efficient. As a result, SincNet’s authors claim that it converges faster and performs better than a standard CNN on the raw waveform and requires less training data^27^. Published examples of its usage include speaker recognition^27^, speech recognition^28^, speaker counting^29^, speaker diarisation^30^, as well as usage in unrelated fields such as induction motor fault diagnosis^31^. A repository by SincNet’s authors^32^ provides open-source Python code, relying on libraries including PyTorch^33^ and Soundfile^34^ to perform speaker recognition.

This study used NIPS4Bplus as training and testing data. NIPS4Bplus is a bioacoustic dataset containing audio files and associated detailed (rich) labels^35^ that is ideal for supervised machine learning classification tasks^15^. The audio files correspond to the training set of the 2013 Neural Information Processing Scaled for Bioacoustics (NIPS4B) challenge for bird song classification^23^ (2013 Bird Challenge). The labels in the original challenge dataset state the presence of bird species for a given audio file but not their precise location in the file. To enhance the usability of the data for machine learning purposes the authors of NIPS4Bplus reviewed each file individually, expanding on the original labels, to generate species tags with detailed temporal annotations^15^.

The classification of bioacoustic sounds according to the species that generated them is a similar task to the one originally performed by SincNet; the recognition of individual speakers in human speech^27^. This study uses the SincNet code for this speaker recognition task^32^ to train a classification model using the NIPS4Bplus bioacoustic dataset. Initially, we maintained the default parameters where possible and did not attempt to enhance the performance of the algorithm in order to facilitate the reproducibility of our results. Later, to evaluate the performance of SincNet and to compare against other techniques, we identified the best performing settings via parameter optimisation. We compared these results with those obtained using: (1) standard CNN models trained directly on the raw waveform without the sinc-based filters; we will refer to them as: “waveform + CNN”; and (2) pre-trained models through transfer learning on extracted features^14^. The waveform + CNN models maintain the same network architecture as SincNet but a standard convolution replaces the first sinc-based convolution^27^. In the pre-trained models, deep learning models trained on large generic image datasets are repurposed through transfer learning by only training the final layers. Three pre-trained models are tested: DenseNet121, ResNet50 and VGG16. They are fine-tuned using sound transformations and feature extraction that convert the labelled sounds into image-like arrays.

## Results

**Figure 1﻿:**
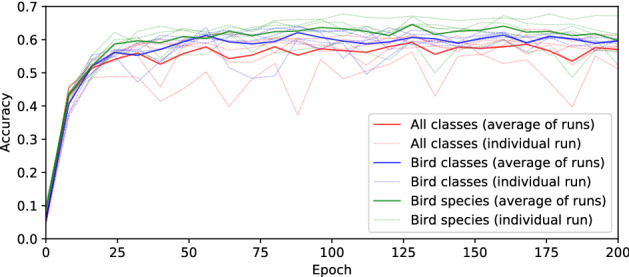
Accuracy over training epochs for 5 replicated training runs from initial models using default SincNet settings. Different colours indicate different tag selections: “All Classes” include insects, one amphibian, birds and their call type (87 classes), “Bird Classes” include only birds and their call type (77 classes), “Bird Species” does not differentiate by call type (51 classes).

Training SincNet on NIPS4Bplus files using default settings yielded rapid results, with accuracy increasing over the first few epochs and surpassing 65% in some of the training runs (Fig. 1). Calculations of receiver operating characteristic (ROC) area under the curve (AUC) averaged 75.6% over 30 trained models, while the accuracy over the same models averaged 60%. Training over 200 epochs took an average of 3.5 h using an NVIDIA GeForce RTX2060 GPU. The variability between training runs (Fig. 1) reflects: the stochastic nature of the training process; the use of different random splits of train and test datasets for each run; and the selection of different classes.

**Figure 2:**
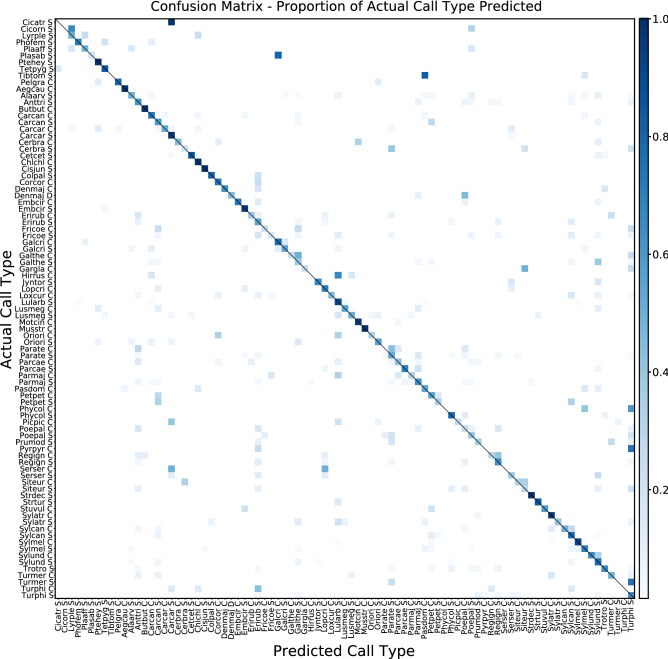
Example of confusion matrix by call type for all classes (87) from an initial model using default SincNet settings. Sound types are sorted first by taxonomic group (insects, amphibian and birds) and then by alphabetical order of their abbreviated scientific name and call type (“S”, “C” and “D” for Song, Call and Drumming). The diagonal line represents successful classifications.

The confusion matrix (Fig. 2) offers an overview of the predictive value of one of the trained models using default settings. Each cell represents the proportion of predictions for each actual call type over the test set. High probabilities aligned with the diagonal indicate successful predictions. All predictions in cells not in the diagonal are incorrect predictions, but these often have lower probabilities. There are sound classes that lack any correct predictions while some predicted classes are repeatedly assigned to multiple, incorrect, classes. Some of these errors may be explained by the characteristics of the data, such as the lesser or greater presence of particular sound types in the dataset (Fig. 5). As a result, predictions are biased towards more abundant call types.

Table 1 presents results for SincNet after hyperparameter tuning and comparative results for other techniques. It shows the performance metrics and other information for the best performing models. The detailed parameters and additional metrics of each model are provided as Supplementary Information 1.

**Table 1:** Performance metrics, running time and parameter sizes of selected models, grouped by tag selection and then sorted by accuracy.

Model	Accuracy	ROC AUC	Precision	Recall	Top 3 accuracy	Top 5 accuracy	Training time (h)	Trainable parameters
**All classes**								
DenseNet121	0.7484	0.7971	0.7639	0.7544	0.9562	0.9759	5.1	7.0 × 10^6^
ResNet50	0.7403	0.7897	0.7562	0.7441	0.9555	0.9752	4.4	23.7 × 10^6^
SincNet	0.7301	0.7562	0.7489	0.7301	0.8993	0.9329	2.1	2.6 × 10^6^
VGG16	0.7294	0.7723	0.7346	0.7331	0.9387	0.9643	6.0	134.6 × 10^6^
Waveform + CNN	0.7017	0.7425	0.7216	0.7017	0.8818	0.9263	1.9	2.5 × 10^6^
**Bird classes**								
ResNet50	0.7674	0.8129	0.7692	0.7674	0.9545	0.9727	4.3	23.7 × 10^6^
DenseNet121	0.7545	0.8290	0.7630	0.7632	0.9462	0.9659	4.9	7.0 × 10^6^
VGG16	0.7530	0.7935	0.7593	0.7553	0.9462	0.9606	5.8	134.6 × 10^6^
SincNet	0.7447	0.7662	0.7625	0.7447	0.8970	0.9327	2.0	2.5 × 10^6^
Waveform + CNN	0.7205	0.7593	0.7348	0.7205	0.8909	0.9273	1.8	2.5 × 10^6^
**Bird species**								
ResNet50	0.7689	0.8046	0.7645	0.7689	0.9629	0.9765	4.4	23.6 × 10^6^
DenseNet121	0.7659	0.8100	0.7664	0.7659	0.9614	0.9765	4.9	7.0 × 10^6^
VGG16	0.7598	0.8174	0.7613	0.7633	0.9568	0.9758	5.8	134.5 × 10^6^
SincNet	0.7356	0.7485	0.7481	0.7356	0.9023	0.9447	1.9	2.5 × 10^6^
Waveform + CNN	0.7091	0.7618	0.7285	0.7091	0.8977	0.9492	1.8	2.4 × 10^6^

## Discussion

The results presented here demonstrate the conceptual simplicity and viability of training a deep learning algorithm using raw audio waveforms for bioacoustic classification. Processing bioacoustic data has typically relied on the initial extraction of features from the audio signal, which are often processed further into handcrafted features prior to their use in machine learning. Researchers in the field are aware of the limitations of this approach, as the most widely used handcrafted features are designed for human speech tasks and focus on spectral characteristics of the sound that may differ from the intended bioacoustic tasks^18,19^. However, traditional deep learning techniques applied directly to the raw waveform have remained largely out of reach, due to limitations primarily associated with insufficient volumes of labelled data. SincNet offers a ready alternative to feature extraction and processing by relying on learnable parametric filters. This architecture, as demonstrated in the results of this study, successfully trains models for a bioacoustic task which converge rapidly and require comparatively small amounts of data.

The ability to bypass feature extraction is recognised as a significant advance in human speech processing^22^. In bioacoustics this may have added significance as it altogether bypasses the selection and use of extracted features that may not be ideal or that introduce bias. Elements of the sound that are valuable for a particular bioacoustic task may be lost through the feature extraction or the selection process. This is not the case when training models on the raw waveform and allowing the algorithm to select the elements of the sound that are best suited for the task. Therefore, we consider that algorithms trained on the raw waveform may simplify the classification process and are likely to be a valuable tool in bioacoustics.

Our initial results using default SincNet settings, without any optimisation, provided useful and relatively accurate results. SincNet models trained on NIPS4Bplus yielded accuracies averaging 60%. Generally, the accuracy decreases and is more variable as the number of classes included in the model increases and the task becomes more complex (Fig. 1). The results using enhanced parameters are broadly similar to those obtained using extracted features on pre-trained models and suggest that SincNet is a useful tool not only for human speech processing but also the classification of bioacoustic sounds. Our Sincnet and waveform + CNN results demonstrate an accuracy consistent with established techniques but with lower training parameters and faster training than pre-trained models (Table 1), two indicators of increased efficiency. Using the same hardware throughout the experiments, the SincNet models took around half the time to train than fine-tuning the fastest pre-trained models. Whilst many factors contribute to this speed difference, a significant one is the processing costs of sound transformations, undertaken by the CPU for the pre-trained models, that are not required in SincNet when working from the raw sound waveform. The greater efficiency also takes place at evaluation time, when models generate predictions for new data, and speeds up the processing of large sound datasets. None of the models obtained accuracies above 80%, suggesting possible classification limits due to the complexity of the data. Although drawing training and test sets from the same data pool is known to simplify the task^25^, these results possibly reflect the difficulty in classifying individually tagged calls from real field recordings, as discussed in the introduction.

**Figure 3:**
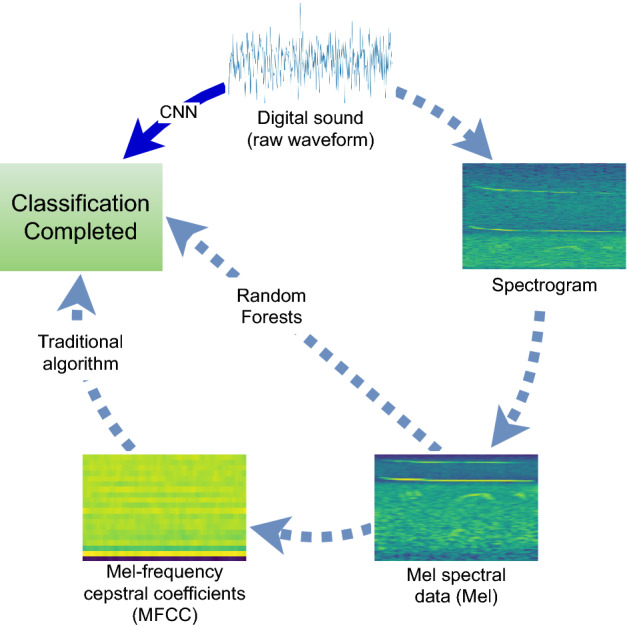
Sound Processing routine. A traditional approach requires four steps from sound to classification (clockwise). The MFCC step may be avoided when using Random Forests^19^. Future routines based only on CNN may process the sound directly (counterclockwise step).

The waveform + CNN models provided classification results that were generally comparable with the other models. This highlights the ability of neural networks to learn from data (raw waveforms) that have traditionally been considered too complex, to have too high a level of dimensionality, to be useful for machine learning. In the past, limited computing capabilities required the simplification of bioacoustic sounds (i.e. reduction in dimensionality) through multiple steps to facilitate classification (Fig. 3). The extraction of Mel-frequency cepstral coefficients from Mel spectral data is a common procedure to reduce the dimensionality of the data, but researchers have raised concerns about information loss, such as semantic information, which could reduce classification performance^19^. Around 2014 researchers proposed abandoning this step as enhanced machine learning algorithms (random forests) could handle the higher-dimensionality data, and using the Mel without the conversion to coefficients preserves more of the original sound information that may aid in the classification task^19^. Results presented here indicate the next logical step in this progression, and suggest that in the future we can directly process the highest-dimensionality data (the raw waveform) with deep learning algorithms and dispense with feature extractions (Mel) and transformations (spectrogram) altogether. This approach eliminates the possible risk of losing relevant information and allows the algorithms to select the elements of the sound that best suit the classification task. It also facilitates the use of classification algorithms by the end-user, as there is no need to choose the type of transformation or extracted features to enhance performance.

Our results also demonstrate that training SincNet successfully is possible with relatively short, adequately-labelled sound data. This is critical for more widespread usage in bioacoustics where access or the capability to generate extensively labelled datasets is limited^15^. Using only the parts of the original files tagged as bioacoustic sounds in the NIPS4Bplus labels, reduces the original 48 min to only 17 min 56 s of sound (or 13 min 8 s for the birds-only parts of this study). When split into training and testing sets, the algorithm trains some of the models in less than 10 min of sound. We estimate that a single expert annotator can generate detailed labels for this amount of data in only a few days. Despite this relatively small amount of sound and the high number of classes, training converges rapidly producing meaningful results within a few training epochs (Fig. 1).

SincNet has the ability to classify sounds even when different sounds are combined and overlapping^31^. This is valuable in bioacoustics as often different species vocalise simultaneously. For example, as indicated in the experimental setup, over 20% of tagged sounds in the NIPS4Bplus dataset overlap with sounds of other species. A review of trained SincNet model predictions for test files containing more than one species is consistent with this property, as there is no apparent reduction in performance. Over 70% of the predictions fall on one of the overlapping species, with a 60:40 split in those predictions between the tagged and the overlapping species. It is also reflected in the ability to train models on this dataset as 20% of training files contain overlapping sounds. Predictions that fall on the overlapping sound slightly decrease the reported accuracy of this study, as the single prediction is matched against the class tag. If we were to consider correct those predictions that fall on the overlapping species the reported accuracy would increase by an additional 4%. Some allowance for this behaviour may be required in order to minimise false negatives in trained models. Obtaining a unique class prediction for each sound event may not be ideal to detect rare sounds or for datasets containing many overlapping sounds. The classification layer of the models (LogSoftmax) returns class probabilities for each frame and the optimal way to combine these across a sound event may be adapted to a particular task. Instead of obtaining a single class prediction for each event it may be more appropriate to select probabilities above a threshold when trying to detect rare sound events. Or output more than one class per event, when high relative probabilities are assigned to different classes over consecutive frames, as this may indicate overlapping sounds. A detailed frame by frame analysis of model outputs for events with two overlapping classes suggests that this is feasible, as the models consistently assign high relative probabilities to both classes.

There are multiple avenues to optimise the performance of SincNet. This study used the default architecture and both default and enhanced parameters. Other targeted optimisations are likely to improve the performance of the algorithm for bioacoustics processing. Most apparent are modifications to the CNN architecture to suit particular bioacoustic tasks. There are also other changes reflected in the literature that may be useful in the field of bioacoustics, including variations in the filter shape or type, changes in initialisation of the filters, and alternative output layers. The sinc-based filters in the current implementation of SincNet have a rectangular shape, but other shapes (triangular, gammatone, Gaussian) have already improved the performance of SincNet in human speech tasks^36^. Another popular type of filters are Gabor filters used both for human speech^22^ and already satisfactorily tested in bioacoustics^26^. The substitution of the softmax output layer by an AM-softmax improves the performance of SincNet in speaker recognition^37^ but we did not get performance improvements in our tests with the NIPS4Plus dataset.

A significant property of SincNet is the interpretability of the trained filters^28,31^. However, it appears that the latest, more efficient release of SincNet available on the repository^32^, is capable of performing the classification tasks without significant alteration of the sinc-based filters from the default initialization. This is understood to be a different behaviour from earlier, less efficient, releases of SincNet in which the sinc-based filters changed as the model learnt. Therefore, the initialisation of filters is likely to have a direct effect on performance. This study used default settings initialising the filter parameters on the mel scale. Other implementations of SincNet do this randomly^24^. It is possible that initialisations that are targeted to the intended bioacoustic task may result in performance enhancements as observed by other researchers^38^. The lack of significant changes to the filters is consistent with the results obtained using waveform + CNN, while the sinc-based filters increase performance, a standard convolutional layer provides comparable classification results. The models using waveform + CNN are no doubt constrained by using the same basic architecture as SincNet, but this was required for comparative purposes. It is likely that better results may be obtained with enhanced architectures; techniques such as neural architecture search, that automatically select best performing neural network architectures, may assist in this process^26^.

Another important area of development already successfully applied to SincNet is transfer learning^30^. In transfer learning, as discussed previously, an algorithm trained on a larger dataset is repurposed to a smaller dataset by training only the final layers of the CNN on the smaller dataset. The importance of this technique in bioacoustics is highlighted by the difficulty in acquiring large datasets^15^. Transfer learning would allow the development of algorithms trained on larger datasets of related taxonomic groups to be repurposed for its usage with other species in the same taxa. This may be particularly useful when training models to identify rare or threatened species that have limited reference recordings. We tested transfer learning across domains by training speaker identification SincNet models on speech datasets (TIMIT^39^ and LibriSpeech^40^) and then fine tuning those models by only training the classification (final) layer on the NIPS4Bplus dataset. The resulting models, after resampling to normalise sampling rates across datasets, had performances above the default SincNet settings but did not improve over the best performing models. This suggests the feasibility of the approach, although greater similarity between datasets may be required to improve results.

Concerns have been raised about a lack of reproducibility in bioacoustic research that may be impeding progress^41^. The present study uses a published open-source CNN architecture, a publicly-released richly annotated birdsong dataset and additional code made available in a repository^42^ to encourage other researchers to replicate the present results. We hope that the easy repeatability of the experiment may encourage others to try the use of SincNet in their own datasets, and motivate them to explore this new line of bioacoustic research where algorithms learn directly from the raw waveform.

## Experimental setup

This study uses SincNet according to the instructions provided by the authors for its application in a different dataset^32^. This section provides an introduction to SincNet and NIPS4Bplus before detailing the experimental procedure.

### SincNet

The first convolutional layer of a standard CNN trained on the raw waveform learns filters from the data, where each filter has a number of parameters that matches the filter length (Eq. 1).

1 $$y\left[ n \right] = x\left[ n \right] \times f\left[ n \right] = \mathop \sum \limits_{i = 0}^{I - 1} x\left[ i \right] \cdot f\left[ {n - i} \right],$$

where $x\left[ n \right]$ is the chunk of the sound, $f\left[ n \right]$ is the filter of length $I$, and $y\left[ n \right]$ is the filtered output. All the elements of the filter ($i$) are learnable parameters. SincNet replaces $f\left[ n \right]$ with another function $g$ that only depends on two parameters per filter: the lower and upper frequencies of a rectangular bandpass filter (Eq. 2).

2 $$g\left[ {n,f_{l} ,f_{h} } \right] = 2f_{h} sinc\left( {2\pi f_{h} n} \right) - 2f_{l} sinc\left( {2\pi f_{l} n} \right),$$

where $f_{l} \text{ and } f_{h}$ are the learnable parameters corresponding to the low and high frequencies of the filter and $sinc\left( x \right) = \frac{sin\left( x \right)}{x}$. The function $g$ is smoothed with a Hamming window and the learnable parameters are initialised with given cut-off frequencies in the interval $\left[ {0,\frac{{f_{s} }}{2}} \right]$, where $f_{s}$ is the sampling frequency.

**Figure 4:**
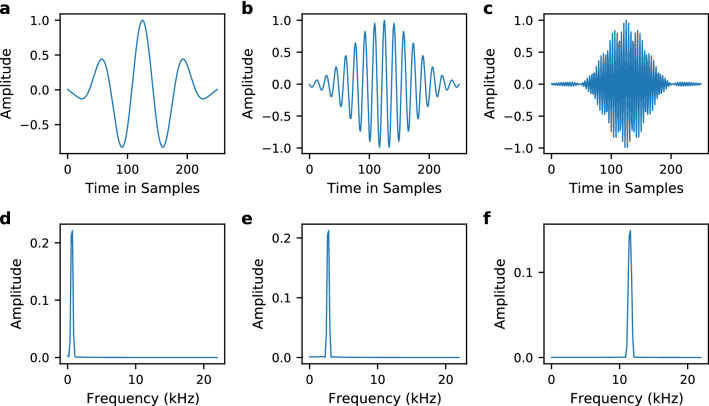
Examples of learned SincNet filters. The top row (**a**–**c**) shows the filters in the time domain, the bottom row (**d**–**f**) shows their respective frequency response.

This first layer of SincNet performs the sinc-based convolutions for a set number and length of filters, over chunks of the raw waveform of given window size and overlap. A conventional CNN architecture follows the first layer, that in this study maintains the architecture and uses both standard and enhanced settings. The standard settings used are those of the TIMIT speaker recognition experiment^27,32^. They include two convolutional layers after the first layer with 60 filters of length 5. All three convolutions use layer normalisation. Next, three fully-connected (leaky ReLU) layers with 2048 neurons each follow, normalised with batch normalisation. To obtain frame-level classification, a final softmax output layer, using LogSoftmax, provides a set of posterior probabilities over the target classes. The classification for each file derives from averaging the frame predictions and voting for the class that maximises the average posterior. Training uses the RMSprop optimiser with the learning rate set to 0.001 and minibatches of size 128. A sample of sinc-based filters generated during this study shows their response both in the time and the frequency domains (Fig. 4).

The SincNet repository^32^ provides an alternative set of settings used in the Librispeech speaker recognition experiment^27^. Tests of the alternative settings, which include changes in the hidden CNN layers, provided similar results to those of the TIMIT settings and are included as Supplementary Information 1.

### NIPS4Bplus

NIPS4Bplus includes two parts: sound files and rich labels. The sound files are the training files of the 2013 NIPS4B challenge for bird song classification^23^. They are a single channel with a 44.1 kHz sampling rate and 32 bit depth. They comprise field recordings collected from central and southern France and southern Spain^15^. There are 687 individual files with lengths from 1 to 5 s for a total length of 48 min. The tags in NIPS4Bplus are based on the labels released with the 2013 Bird Challenge but annotated in detail by an experienced bird watcher using dedicated software^15^. The rich labels include the name of the species, the class of sound, the starting time and the duration of each sound event for each file. The species include 51 birds, 1 amphibian and 9 insects. For birds there can be two types of vocalisations: call and song; and there is also the drumming of a woodpecker. Calls are generally short sounds with simple patterns, while songs are usually longer with greater complexity and can have modular structures or produced by one of the sexes^8,13^. In the dataset, only bird species have more than one type of sound, with a maximum of two types. The labels in NIPS4Bplus use the same 87 tags present in the 2013 Bird Challenge training dataset with the addition of two other tags: “human” and “unknown” (for human sounds and calls which could not be identified). Tagged sound events in the labels typically correspond to individual syllables although in some occasions the reviewer included multiple syllables into single larger events^15^. The tags cover only 569 files of the original training set of 687 files. Files without tags include 100 that, for the purpose of the challenge, had no bird sounds but only background noise. Other files were excluded for different reasons such as vocalisations hard to identify or containing no bird or only insect sounds^15^. The 2013 Bird Challenge also includes a testing dataset with no labels that we did not use^15^.

**Figure 5:**
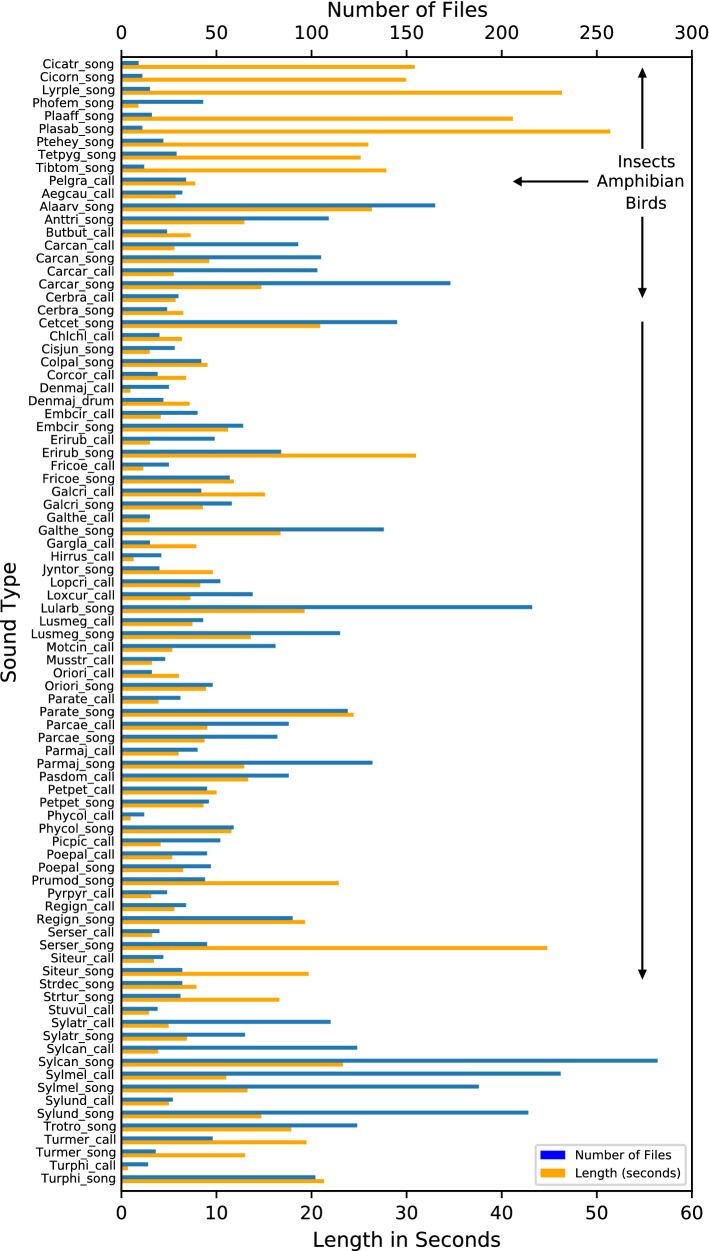
Distribution of sound types by number of calls (number of files) and total length in seconds. Sound types are sorted first by taxonomic group and then by alphabetical order.

The total number of individual animal sounds tagged in the NIPS4Bplus labels is 5478. These correspond to 61 species and 87 classes (Fig. 5). The mean length of each tagged sound ranges from ~ 30 ms for Sylcan_call (the call of *Sylvia cantillans*, subalpine warbler) to more than 4.5 s for Plasab_song (the song of *Platycleis sabulosa,* sand bush-cricket). The total recording length for a species ranges from 0.7 s for Turphi_call (the call of *Turdus philomelos*, song thrush) to 51.4 s for Plasab_song. The number of individual files for each call type varies greatly from 9 for Cicatr_song (the call of C*icadatra atra*, black cicada) to 282 for Sylcan_call.

### Processing NIPS4Bplus

The recommended pre-processing of human speech files for speaker recognition using SincNet includes the elimination of silent leading and trailing sections and the normalisation of the amplitude^27^. This study attempts to replicate this by extracting each individual sound as a new file according to the tags provided in the NIPS4Bplus labels. A Python script^42^ uses the content of the labels to read each wavefile, apply normalisation, select the time of origin and length specified in each individual tag and save it as a new wavefile. The name of the new file includes the original file name and a sequential number suffix according to the order in which tags are listed in the label files (the start time of the sound) to match the corresponding call tags at the time of processing. Each wavefile in the new set fully contains a sound according to the NIPS4Bplus labels. A cropped file may contain sounds from more than one species^15^, with over 20% of the files in the new set overlapping, at least in part, with sound from another species. The machine learning task does not use files containing background noise or the other parts of the files that are not tagged in the NIPS4Bplus labels. A separate Python script^42^ generates the lists of files and tags that SincNet requires for processing. The script randomly generates a 75:25 split into lists of train and test files and a Python dictionary key that assigns each file to the corresponding tag according to the file name. The script selects only files confirmed as animal sounds (excluding the tags “unknown” and “human”) and generates three different combinations of tags, as follows: (1) “All classes”: includes all the 87 types of tags originally included in the 2013 Bird Challenge training dataset; (2) “Bird classes”: excludes tags for insects and one amphibian species for a total of 77 classes; and (3) “Bird species”: one class for each bird species independently of the sounds type (call, songs and drumming are merged for each species) for a total of 51 classes. The script also excludes three very short files (length shorter than 10 ms) which could not be processed without code modifications.

To facilitate the repeatability of the results, this study attempts to maintain the default parameters of SincNet used in the TIMIT speaker identification task^27,32^. The number and length of filters in the first sinc-based convolutional layer was set to the same values as the TIMIT experiment (80 filters of length 251 samples) as was the architecture of the CNN. The filters were initialised following the Mel scale cut-off frequencies. We did change the following parameters: (1) reduced the window frame size (cw_len) from 200 to 10 ms to accommodate for the short duration of some of the sounds in the NIPS4Bplus tags (such as some bird vocalisations); (2) reduced the window shift (cw_shift) from 10 to 1 ms in proportion to the reduction in window size (a value a 0.5 could not be given without code modifications); (3) updated the sampling frequency setting (fs) from the TIMIT 16,000 to the 44,100 Hz of the present dataset; and (4) updated the number of output classes (class_lay) to match the number of classes in each training run.

To evaluate performance, the training sequence was repeated with the same settings and different random train and test file splits. Five training runs took place for each of the selection of tags: “All classes”, “Bird classes” and “Bird species”.

### Enhancements and comparisons

Changes in the parameters of SincNet result in different levels of performance. To assess possible improvements and provide baselines to compare against other models we attempted to improve the performance by adjusting a series of parameters, but did not modify the number of layers or make functional changes to the code other than the two outlined below. The parameters tested include: the length of the window frame size, the number and length of the filters in the first layer, number of filters and lengths of the other convolutional and fully connected layers, the length and types of normalisation in the normalisation layers, alternative activation and classification functions, and the inclusion of dropouts (Supplementary Information 1). In addition the SincNet code includes a hard-coded random amplification of each sound sequence; we also tested changing the level and excluding this random amplification through changes in the code. In order to process window frames larger than some of the labelled calls in the NIPS4Bplus dataset, the procedure outlined earlier in which files are cut according to the labels was replaced by a purpose-built process. The original files were not cut, instead a custom python script^42^ generated train and test file lists that contain the start and length of each labelled call. A modification of the SincNet code^42^ uses these lists to read the original files and select the labelled call. When the call is shorter than the window frame the code randomly includes the surrounding part of the file to complete the length of the window frame. Grid searches for individual parameters or combinations of similar parameters, over a set number of epochs, selected the best performing values. We also tested the use of the Additive Margin Softmax (AM-softmax) as a cost function^37^. The best performing models reported in the results use combinations of the best parameter values (Supplementary Information 1). All enhancements and model comparisons use the same dataset selection, that is the same train and test dataset split, of the normalised files for each set of tagged classes.

The comparison using waveform + CNN models trained directly on the raw waveform, replaces the initial sinc-based convolution of SincNet with a standard 1d convolutional layer^27^, thus retaining the same network architecture as SincNet. As with SincNet enhancements, a series of parameter searches provided the best parameter combinations to obtain the best performing models.

The pre-trained models used for comparison are DenseNet121, ResNet50 and VGG16 with architectures and weights sourced from the Torchvision library of PyTorch^33^. We tested three types of spectrograms: Fast Fourier Transform (FFT), Mel spectrum (Mel) and Mel-frequency cepstral coefficient (MFCC) to fine-tune the pre-trained models. FFT calculations used a frame length of 1024 samples, 896 samples overlap and a Hamming window. Mel spectrogram calculations used 128 Mel bands. Once normalised and scaled to 255 pixel intensity three repeats of the same spectrogram represented each of the three input channels of the pre-trained models. The length of sound used to generate the spectrograms was 3 s, and similarly with routines above, for labelled calls shorter than 3 s the spectrogram would randomly include the surrounding sounds. That is, the extract would randomly start in the interval between the end of the labelled call minus 3 s and the start of the call plus 3 s. This wholly includes the labelled call but its position is random within the 3 s sample. A fully connected layer replaced the final classifying layer of the pre-trained models to output the number of labelled classes. In the fine-tuning process the number of trainable layers of the model was not limited to the final fully connected layer, but also included an adjustable number of final layers to improve the results. The learning rate set initially to 0.0001 was halved if the validation loss stopped decreasing for 10 epochs.

### Metrics

Measures of performance include accuracy, ROC AUC, precision, recall, F1 score, top 3 accuracy and top 5 accuracy. Accuracy, calculated as part of the testing routine, is the ratio between the number of correctly predicted files of the test set and the total number of test files. The calculation of the other metrics uses the Scikit-learn module^43^ relying on the predicted values provided by the model and performing weighted averages. The ROC AUC calculation uses the mean of the posterior probabilities provided by SincNet for each tagged call. In the pre-trained models the ROC AUC calculations used the probabilities obtained after normalising the output with a softmax function.

## Supplementary Information

Supplementary Information.
