# Supplementary material for: Bioacoustic classification of avian calls from raw sound waveforms with an open-source deep learning architecture
Source: Sci Rep. 2021 Aug 3;11:15733. doi: 10.1038/s41598-021-95076-6 (PMC8333097; doi:10.1038/s41598-021-95076-6)
Supplement: Supplementary file 1 — Supplementary Information. [file 41598_2021_95076_MOESM1_ESM.pdf]

Bioacoustic Classification of Avian Calls from Raw Sound Waveforms with an Open-source Deep Learning Architecture

Francisco J. Bravo Sanchez, Md Rahat Hossain, Nathan B. English & Steven T. Moore

SUPPLEMENTARY INFORMATION

This document includes the following: model parameters, additional metrics and parameters tested during SincNet hyperparameter tuning.

1. Model Parameters

Tables S1 to S6 detail the parameters used for each model or type of model presented in the main text. They are grouped by model type: SincNet Default (Timit and Librispeech), SincNet Enhanced, Waveform+CNN and pre-trained models. Detail parameters are provided for each tag class selection (all classes, bird classes and bird species) but not for SincNet Default as only class_lay differs between class selections.

Both the SincNet and Waveform+CNN models use the SincNet code and the parameters are presented with the same nomenclature used in SincNet configuration files (.cfg) except for the amplification factor which is hard-coded and not included in the configuration. We maintain this nomenclature for the pre-trained models (tables S4 to S6) when they use the same parameters.

The size of each SincNet training epoch is controlled by the size of each random minibatch (batch_size) and the number of batches (N_batches), and is independent of the size of the training dataset. In the pre-trained models the size of each epoch corresponds to the size of the training dataset to a maximum of 4110 files (the size of the training dataset is provided in tables S4 to S6). Therefore the number of files processed over each epoch is greater in SincNet than in the pre-trained models.

Note that, as indicated in the main text, SincNet code assigns the function LogSoftmax to the configuration selection of softmax (such as class_act).

SincNet Default		
**Parameter**	**Timit**	**Librispeech**
[windowing]		
fs	44100	44100
cw_len	10	10
cw_shift	1	1
fact_amp	0.2 (default hard coded)	0.2 (default hard coded)
[cnn]		
cnn_N_filt	80,60,60	80,60,60
cnn_len_filt	251,5,5	251,5,5
cnn_max_pool_len	3,3,3	3,3,3
cnn_use_laynorm_inp	True	True
cnn_use_batchnorm_inp	False	False
cnn_use_laynorm	True,True,True	True,True,True
cnn_use_batchnorm	False,False,False	False,False,False
cnn_act	leaky_relu,leaky_relu, leaky_relu	relu,relu,relu
cnn_drop	0.0,0.0,0.0	0.0,0.0,0.0
[dnn]		
fc_lay	2048,2048,2048	2048,2048,2048
fc_drop	0.0,0.0,0.0	0.0,0.0,0.0
fc_use_laynorm_inp	True	True
fc_use_batchnorm_inp	False	False
fc_use_batchnorm	True,True,True	True,True,True
fc_use_laynorm	False,False,False	False,False,False
fc_act	leaky_relu,leaky_relu,leaky_relu	leaky_relu,linear,leaky_relu
[class]		
class_lay	All Classes - 87, Bird Classes - 77, Bird Species - 51	
class_drop	0.0	0.0
class_use_laynorm_inp	False	True
class_use_batchnorm_inp	False	False
class_use_batchnorm	False	False
class_use_laynorm	False	False
class_act	softmax	softmax
[optimization]		
lr	0.001	0.001
batch_size	128	128
N_epochs	200	200
N_batches	800	800
N_eval_epoch	8	8
seed	1234	1234

Table S1: Training parameters for SincNet models using default configurations

SincNet Enhanced			
**Parameter**	**All Classes**	**Bird Classes**	**Bird Species**
[windowing]			
fs	44100	44100	44100
cw_len	18	16	16
cw_shift	1	1	1
fact_amp	0	0	0
[cnn]			
cnn_N_filt	220,60,60	220,60,60	220,60,60
cnn_len_filt	151,5,5	151,5,5	151,5,5
cnn_max_pool_len	5,5,5	5,5,5	5,5,5
cnn_use_laynorm_inp	False	False	False
cnn_use_batchnorm_inp	False	False	False
cnn_use_laynorm	False,False,False	False,False,False	False,False,False
cnn_use_batchnorm	True,True,True	True,True,True	True,True,True
cnn_act	leaky_relu,leaky_relu, leaky_relu	leaky_relu,leaky_relu, leaky_relu	leaky_relu,leaky_relu, leaky_relu
cnn_drop	0.0,0.0,0.0	0.0,0.0,0.0	0.0,0.0,0.0
[dnn]			
fc_lay	1024,1024,1024	1024,1024,1024	1024,1024,1024
fc_drop	0.0,0.0,0.0	0.0,0.0,0.0	0.0,0.0,0.0
fc_use_laynorm_inp	False	False	False
fc_use_batchnorm_inp	False	False	False
fc_use_batchnorm	True,True,True	True,True,True	True,True,True
fc_use_laynorm	False,False,False	False,False,False	False,False,False
fc_act	relu,relu,relu	relu,relu,relu	relu,relu,relu
[class]			
class_lay	87	77	51
class_drop	0.0	0.0	0.0
class_use_laynorm_inp	False	False	False
class_use_batchnorm_inp	False	False	False
class_use_batchnorm	False	False	False
class_use_laynorm	False	False	False
class_act	softmax	softmax	softmax
[optimization]			
lr	0.001	0.001	0.001
batch_size	128	128	128
N_epochs	400	400	400
N_batches	80	80	80
N_eval_epoch	8	8	8
seed	1234	1234	1234

Table S2:Training parameters for enhanced SincNet models

Waveform+CNN			
**Parameter**	**All Classes**	**Bird Classes**	**Bird Species**
[windowing]			
fs	44100	44100	44100
cw_len	14	14	10
cw_shift	1	1	1
fact_amp	0	0	0
[cnn]			
cnn_N_filt	210,60,60	210,60,60	210,60,60
cnn_len_filt	151,5,5	151,5,5	151,5,5
cnn_max_pool_len	5,5,5	5,5,5	5,5,5
cnn_use_laynorm_inp	False	False	False
cnn_use_batchnorm_inp	False	False	False
cnn_use_laynorm	False,False,False	False,False,False	False,False,False
cnn_use_batchnorm	True,True,True	True,True,True	True,True,True
cnn_act	leaky_relu,leaky_relu, leaky_relu	leaky_relu,leaky_relu, leaky_relu	leaky_relu,leaky_relu, leaky_relu
cnn_drop	0.0,0.0,0.0	0.0,0.0,0.0	0.0,0.0,0.0
[dnn]			
fc_lay	1024,1024,1024	1024,1024,1024	1024,1024,1024
fc_drop	0.0,0.0,0.0	0.0,0.0,0.0	0.0,0.0,0.0
fc_use_laynorm_inp	False	False	False
fc_use_batchnorm_inp	False	False	False
fc_use_batchnorm	True,True,True	True,True,True	True,True,True
fc_use_laynorm	False,False,False	False,False,False	False,False,False
fc_act	leaky_relu,leaky_relu, leaky_relu	leaky_relu,leaky_relu, leaky_relu	leaky_relu,leaky_relu, leaky_relu
[class]			
class_lay	87	77	51
class_drop	0.0	0.0	0.0
class_use_laynorm_inp	False	False	False
class_use_batchnorm_inp	False	False	False
class_use_batchnorm	False	False	False
class_use_laynorm	False	False	False
class_act	softmax	softmax	softmax
[optimization]			
lr	0.001	0.001	0.001
batch_size	128	128	128
N_epochs	400	400	400
N_batches	80	80	80
N_eval_epoch	4	4	4
seed	1234	1234	1234

Table S3:Training parameters for waveform + CNN models

DenseNet121			
**Parameter**	**All Classes**	**Bird Classes**	**Bird Species**
fs	44100	44100	44100
cw_len	3000	3000	3000
fact_amp	0	0	0
Sound features	Mel	Spectrogram	Mel
lr (initial)	0.0001	0.0001	0.0001
batch_size	10	10	10
shuffle	True	True	True
N_epochs	50	50	50
class_lay	87	77	51
% trainable layers	30%	30%	30%
Size of training dataset	4110	3959	3959

Table S4:Training parameters for pre-trained DenseNet121 models

ResNet50			
**Parameter**	**All Classes**	**Bird Classes**	**Bird Species**
fs	44100	44100	44100
cw_len	3000	3000	3000
fact_amp	0	0	0
Sound features	Mel	Mel	Mel
lr (initial)	0.0001	0.0001	0.0001
batch_size	20	20	20
shuffle	True	True	True
N_epochs	50	50	50
class_lay	87	77	51
% trainable layers	34%	34%	34%
Size of training dataset	4110	3959	3959

Table S5:Training parameters for pre-trained ResNet50 models

VGG16			
**Parameter**	**All Classes**	**Bird Classes**	**Bird Species**
fs	44100	44100	44100
cw_len	3000	3000	3000
fact_amp	0	0	0
Sound features	Mel	Spectrogram	Mel
lr (initial)	0.0001	0.0001	0.0001
batch_size	5	5	5
shuffle	True	True	True
N_epochs	50	50	50
class_lay	87	77	51
% trainable layers	32%	32%	32%
Size of training dataset	4110	3959	3959

Table S6:Training parameters for pre-trained VGG16 models

2. Additional Metrics

2.1. Metrics for Selected Models

Table S7 lists additional metrics for selected models. It repeats some of the metrics included in Table 1 of the main text to provide context. The calculation of false positive rates and false negative rates is weighted across classes. “ROC AUC Mean Exp” provides an alternative calculation of the ROC AUC. The original ROC AUC calculation uses probabilities obtained by averaging for each tagged call, the LogSoftmax output of each frame (a logarithmic value). The alternative “ROC AUC Mean Exp” calculates the exponential of the LogSoftmax output for each frame before averaging across the tag. These values are provided both for SincNet and Waveform + CNN models.

Model	Accuracy	ROC AUC	Precision	Recall	F1	False Positive Rate	False Negative Rate	ROC AUC Mean Exp
All classes								
DenseNet121	0.7484	0.7971	0.7639	0.7544	0.7404	0.0044	0.2233	-
ResNet50	0.7403	0.7897	0.7562	0.7441	0.7277	0.0050	0.2105	-
SincNet	0.7301	0.7562	0.7489	0.7301	0.7231	0.0062	0.2303	0.7591
VGG16	0.7294	0.7723	0.7346	0.7331	0.7182	0.0043	0.2336	-
Waveform + CNN	0.7017	0.7425	0.7216	0.7017	0.6889	0.0075	0.2376	0.7669
Bird classes								
ResNet50	0.7674	0.8129	0.7692	0.7674	0.7582	0.0045	0.2008	-
DenseNet121	0.7545	0.8290	0.7630	0.7632	0.7398	0.0044	0.2096	-
VGG16	0.7530	0.7935	0.7593	0.7553	0.7456	0.0042	0.2283	-
SincNet	0.7447	0.7662	0.7625	0.7447	0.7408	0.0057	0.2219	0.7865
Waveform + CNN	0.7205	0.7593	0.7348	0.7205	0.7125	0.0066	0.2373	0.7783
Bird species								
ResNet50	0.7689	0.8046	0.7645	0.7689	0.7554	0.0071	0.1925	-
DenseNet121	0.7659	0.8100	0.7664	0.7659	0.7583	0.0077	0.2196	-
VGG16	0.7598	0.8174	0.7613	0.7633	0.7505	0.0073	0.2175	-
SincNet	0.7356	0.7485	0.7481	0.7356	0.7408	0.0057	0.2219	0.7865
Waveform + CNN	0.7091	0.7618	0.7285	0.7091	0.7046	0.0109	0.2552	0.7694

Table S7:Additional metrics for selected models, grouped by tag selection and then sorted by accuracy

2.2. Metrics for Default SincNet Models

The SincNet repository provides two sets of default settings for two different datasets: Timit and Librispeech. Table S8 list the metrics for models trained using these settings adapted as indicated in table S1.

Model	Accuracy	ROC AUC	Precision	Recall	Top 3 Accuracy	Top 5 Accuracy	F1	False Positive Rate	False Negative Rate
All classes									
Timit	0.6321	0.7511	0.6707	0.6321	0.8336	0.8920	0.6246	0.0104	0.3094
Librispeech	0.6404	0.7559	0.6713	0.6404	0.8366	0.8942	0.6220	0.0113	0.2714
Bird classes									
Timit	0.6353	0.7577	0.6895	0.6353	0.8226	0.8870	0.6261	0.0149	0.2799
Librispeech	0.6455	0.7494	0.6897	0.6455	0.8280	0.8841	0.6349	0.0125	0.2816
Bird species									
Timit	0.6778	0.7789	0.7087	0.6778	0.8734	0.9257	0.6613	0.0184	0.2500
Librispeech	0.6394	0.7654	0.6766	0.6394	0.8470	0.9167	0.6330	0.0178	0.3004

Table S8:Metrics for SincNet models using default settings

2.3. ROC AUC Plots for SincNet Models

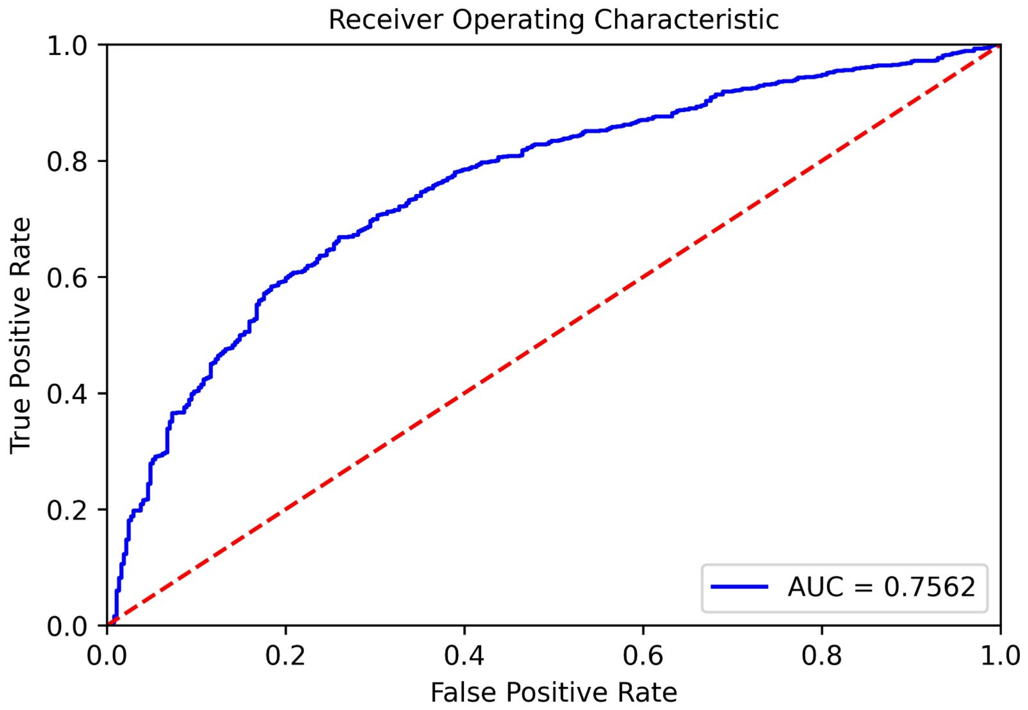

Figure S 1: ROC AUC plot for enhanced SincNet model - All classes

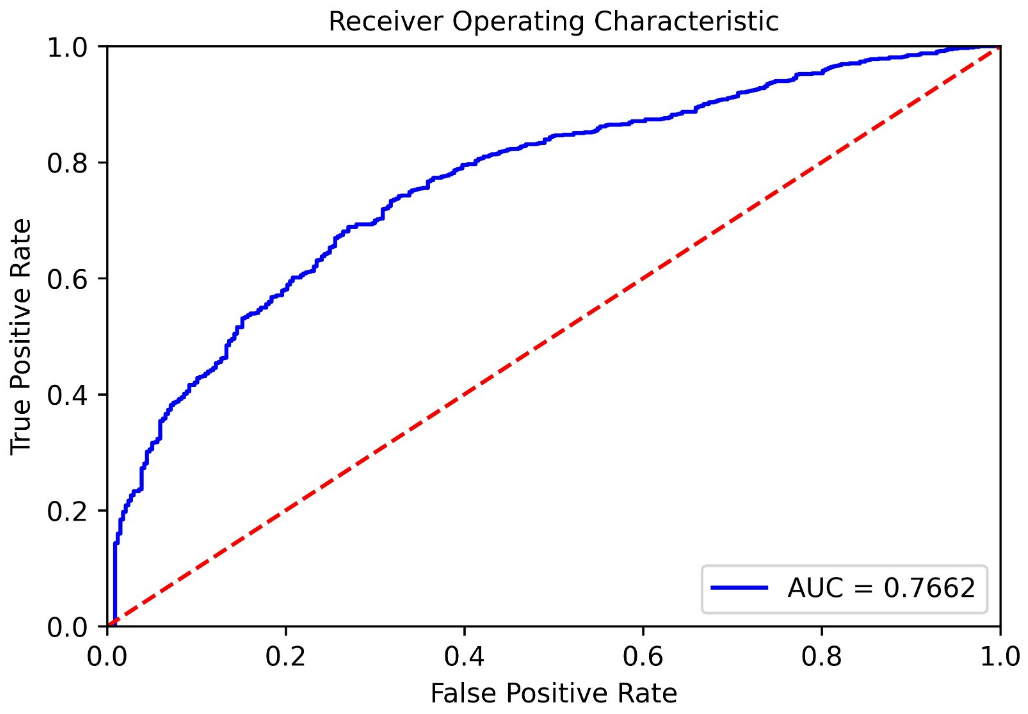

Figure S 2: ROC AUC plot for enhanced SincNet model - Bird classes

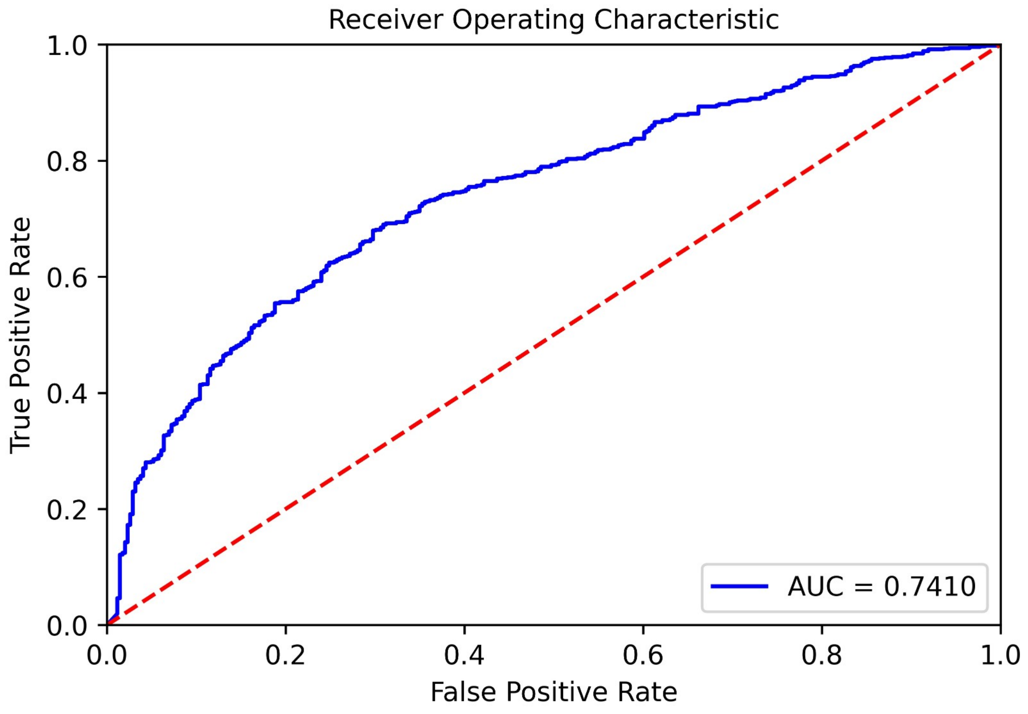

Figure S 3: ROC AUC plot for enhanced SincNet model - Bird species

3. Parameters Tested

Table S 7 lists the parameters tested for hyperparameter tuning. Note that code modifications were required to test some of the values listed in the following parameters: cw_len, fact_amp and class_act.

Parameter	Values Tested
[windowing]	
cw_len	8, 8.5, 9, 9.5, 10, 11, 12, 13, 14, 15, 16, 17, 18, 19, 20, 21, 22, 23, 24, 25, 26, 27, 28, 29, 30, 35, 40, 45, 50
fact_amp	0, 0.1, 0.2, 0.3
[cnn]	
cnn_N_filt	[70,50,50], [80,40,40], [80,50,50], [90,60,60], [90,80,80], [100,50,50], [100,60,60], [110,50,50], [110,60,60], [120,50,50], [120,60,60], [130,60,60], [140,60,60], [150,60,60], [160,60,60], [170,60,60], [180,60,60], [190,60,60], [200,60,60], [210,60,60], [220,60,60], [230,50,50], [230,60,60], [230,70,70], [250,60,60], [260,60,60], [270,60,60], [280,60,60], [290,60,60], [300,60,60]
cnn_len_filt	[51,4,4], [51,5,5], [101,5,5], [121,6,6], [126,4,4], [126,5,5], [126,6,6], [151,4,4], [151,5,5], [151,6,6], [151,7,7], [151,8,8], [176,5,5], [176,6,6], [251,4,4], [251,6,6], [351,5,5]
cnn_max_pool_len	[2,2,2], [3,3,3], [4,4,4], [4,5,6], [5,5,5]
cnn_use_laynorm_inp	False, True
cnn_use_batchnorm_inp	False
cnn_use_laynorm	[False,False,False], [True,True,True]
cnn_use_batchnorm	[False,False,False], [True,True,True]
cnn_act	[leaky_relu,leaky_relu,leaky_relu], [relu,relu,relu], [sigmoid,sigmoid,sigmoid], [tanh,tanh,tanh]
cnn_drop	[0,0,0], [0.1,0.1,0.1], [0.25,0.25,0.25], [0.5,0.5,0.5]
[dnn]	
fc_lay	[1024,1024,1024], [1536,1536,1536], [2048,2048,2048], [3072,3072,3072], [4096,4096,4096]
fc_drop	[0,0,0], [0.1,0.1,0.1], [0.25,0.25,0.25], [0.5,0.5,0.5]
fc_use_laynorm_inp	False, True
fc_use_batchnorm_inp	False
fc_use_batchnorm	[False,False,False], [True,True,True]
fc_use_laynorm	[False,False,False], [True,True,True]
fc_act	[leaky_relu,leaky_relu,leaky_relu], [leaky_relu,linear,leaky_relu], [relu,relu,relu], [sigmoid,sigmoid,sigmoid], [tanh,tanh,tanh]
[class]	
class_drop	0.1, 0.25, 0.4, 0.5
class_use_laynorm_inp	False, True
class_use_batchnorm_inp	False
class_use_batchnorm	False, True
class_use_laynorm	False, True
class_act	[elu,elu,elu], [leaky_relu,leaky_relu,leaky_relu], [logsoftmax,logsoftmax,logsoftmax], [relu,relu,relu], [sigmoid,sigmoid,sigmoid], [softmax,softmax,softmax], [tanh,tanh,tanh]

Table S9:Parameter values tested during SincNet models enhancement
